# Social media analytics of the Internet of Things

**DOI:** 10.1007/s43926-021-00016-5

**Published:** 2021-07-19

**Authors:** Jim A. Scheibmeir, Yashwant K. Malaiya

**Affiliations:** 1 Systems Engineering Department, Colorado State University, 400 Isotope Dr, Fort Collins, CO 80523 USA; 2 Computer Science Department, Colorado State University, 1873 Campus Delivery, Fort Collins, CO 80523 USA

**Keywords:** Internet of Things, Social media, Cybersecurity, Machine learning, Sentiment analysis, Popularity prediction

## Abstract

The Internet of Things technology offers convenience and innovation in areas such as smart homes and smart cities. Internet of Things solutions require careful management of devices and the risk mitigation of potential vulnerabilities within cyber-physical systems. The Internet of Things concept, its implementations, and applications are frequently discussed on social media platforms. This research illuminates the public view of the Internet of Things through a content-based and network analysis of contemporary conversations occurring on the Twitter platform. Tweets can be analyzed with machine learning methods to converge the volume and variety of conversations into predictive and descriptive models. We have reviewed 684,503 tweets collected in a 2-week period. Using supervised and unsupervised machine learning methods, we have identified trends within the realm of IoT and their interconnecting relationships between the most mentioned industries. We have identified characteristics of language sentiment which can help to predict the popularity of IoT conversation topics. We found the healthcare industry as the leading use case industry for IoT implementations. This is not surprising as the current COVID-19 pandemic is driving significant social media discussions. There was an alarming dearth of conversations towards cybersecurity. Recent breaches and ransomware events denote that organizations should spend more time communicating about risks and mitigations. Only 12% of the tweets relating to the Internet of Things contained any mention of topics such as encryption, vulnerabilities, or risk, among other cybersecurity-related terms. We propose an IoT Cybersecurity Communication Scorecard to help organizations benchmark the density and sentiment of their corporate communications regarding security against their specific industry.

## Introduction

The Internet of Things (IoT) is an appealing technology that has eased the management of homes through smart appliances and has enticed industries such as automotive, transportation, and agriculture [1]. IoT was first introduced in 1999 as a technology concept for solving opportunities within logistics [2]. The IoT phenomenon brings compute from the cloud closer to people and things [3]. Today, consumers of data are also producers of data. Twitter users tweet nearly 277,000 times every single minute [4]. The action of liking or retweeting a tweet is yet another data point.

We have collected 684,503 tweets within a two-week period from May 1st, 2021, through May 14th, 2021. Twitter data has been utilized in several recent research investigations [5–7]. Social media platforms have been found to support access to information, discuss and solve engineering problems, identify new trends and communicate science to a public audience [8–12]. We extend the collected Twitter data with metadata using hierarchical clustering techniques and content-based analysis. The clustering algorithm is informed of proper cluster distribution by the within-cluster sum of squares (WSS) and average silhouette methods. A content-based analysis is then performed to identify the number of industries, trends, and technology vendors having a presence in the tweets. Sentiment analysis is carried out for tweets classified towards the industries and vendor technologies. Factors such as the trend labels, industry labels, and sentiment scores are then used in naïve Bayes prediction models. We illustrate the relationships, or lack of, between the trends, industries, and technology providers utilizing network graphs. Section two contains a brief background on the topics of IoT and social media. The research and analysis methodology are described in detail within section three. Finally, the fourth and fifth sections offer discussion and conclusion to the research. The main contributions of this research work include:

- Using advanced statistical and machine learning (ML) methods including naïve Bayes, hierarchical clustering, and natural language processing with sentiment analysis, we evaluate 684,503 contemporary tweets on the topic of the Internet of Things to shed light on public opinion, technology trends, popular industry usage and the popularity and sentiment of technology providers in this space.
- We uncover the substantial problem of a lack of cybersecurity discussion within the IoT tweets. No cybersecurity concepts were identified in the top ten trends. Organizations must increase their cybersecurity communication cadence to meet the risks.
- We analyzed tweets to identify industries where IoT concepts and technology are being discussed. We found healthcare to be the leading industry of mention.
- We propose a new IoT Cybersecurity Communications Scorecard. The scorecard uses a combined index of mention density and sentiment analysis to provide a benchmark of cybersecurity communication posture scores by industry.
- The top three trends identified within the IoT tweets were data science, machine learning and big data. We performed a network analysis to identify relationships between trends and industries, such as what industries have the greatest or least inclusion of trending concepts and technology.
- We evaluate commercial vendors by the sentiment of messages where they are discussed, as well as the volume of mentions. We provide a positional rank of a selection of IoT commercial technologies based upon this analysis.

We believe our research benefits cybersecurity experts, IoT practitioners, and commercial firms. Cybersecurity practitioners and organizational leaders can utilize our findings and scorecard to benchmark areas of their internal behavior. Practitioners, such as developers and engineers of IoT systems, can utilized this research to identify trends within the realm of IoT. Marketing departments of commercial firms benefit from the sentiment analysis and predictive models that shed light on Twitter user behavior regarding the communication of IoT systems. Our contributions are further discussed.

## Background work

### Use of social media in research

The Twitter data has been utilized in several recent research investigations [5–9]. The public availability of the tweets allows researchers to extract valuable conclusions from them [13]. It has been found that the geotagging of twitter users’ tweets can complement surveys as well as enhance a sampling profile [14]. The same study found that their survey showed bias towards elderly participants while the Twitter data was biased towards a younger population. The researchers utilized these conflicting biases to balance their findings. A study of 640 university students found that the leading factor for using social media was to search for and access information [8]. An earlier study by Bougie et al. [9] followed software engineering groups on Twitter to determine how they utilized the platform. This study found that 23% of the groups’ tweets were towards software engineering topics. Of that 23% of their total tweets which regarded software engineering, 62% were towards solving software engineering problems. Another study sought to answer if software engineering practitioners use and cite scientific research in their blogs; they do not [10]. Rather, software engineering practitioners utilize social media to become up to date on technology trends [11]. Another research article states that microblogging serves by linking to web resources, connecting users, and directing users’ attention, as well as offering another channel for the public communication of science [14].

### Related works on the Internet of Things

Implementing an IoT system requires storage, networks, load-balancing, and analysis tools. According to Atalay and Angin [15], an IoT solution should utilize network partitions in private clouds which provide partitioning for enhancing security. Such network partitions could encapsulate the concerns of actuators and sensors, the model of system states, and the business and program logic. An encryption key management system would be utilized to support encryption across network enclaves and an intrusion detection system (IDS) could be implemented to identify malicious activity.

The growing interest in IoT and the implementation of the systems have resulted in large cyber-attack surfaces [15]. A few well-known cyber-physical system attacks include the Stuxnet effect on an Iranian uranium enrichment plant [16] and more recently, a ransomware attack upon Colonial Pipeline that resulted in the gas pipeline being shut down for six days and a near $5 million payout to the hackers [17]. Another recent example of the threat to cyber-physical systems is the Solarwinds attack and the resulting 25% of North American electric utilities that were vulnerable [18]. There are also known consumer exploits including hacked Smart TVs listening to conversations, personal information being extracted from coffee machines, and security cameras leaking images [19]. Today, IoT implementations may be utilized to carry medicine, medical samples, and to assist with the management of pandemics [20], furthering the need for rigor and security in the implementations. In a 2019 research survey of 220 security leaders in industrial and manufacturing, 79% of respondents indicated they had experienced an IoT cyberattack within that past year [21]. The security aspects of IoT have the attention of legitimate organizations who seek to enhance the defense as well as the hackers.

The cybersecurity concerns of IoT systems are growing in complexity and have insufficient security solutions [15]. The evaluation of cyber-physical system component vulnerabilities is a challenging task due to the sheer number of devices and their varied configurations. Common threats include denial of service (DoS) attacks while a common weakness is insecure wireless networking [15]. The complexity of IoT systems and their emergent behavior also complicate the testing of the systems [22].

To manage the complexity while achieving value and providing security of the system’s assets and users, five best practices have been suggested by Shi et al. [3]. Good service management of edge computing and IoT systems include these five considerations:

- Differentiation in device identification to discern specific state metrics such as the health of the specific device instances.
- Extensibility in the system to allow for replacement endpoints being easily swapped in and out.
- Isolation of access and data via roles and other controlling factors.
- Security/privacy which preserves availability and confidentiality characteristics.
- Optimization of the system and components’ attributes such as cost, latency, or bandwidth.

While Shi et al. [3] mention the optimization of system components including latency and bandwidth, Fizza et al. [23] dive deeper into optimization stating that existing definitions of quality of experience (QoE) must be renewed with the autonomous IoT systems in mind. The same research found that if QoE is not considered in autonomous IoT applications, poor quality of decisions and resulting actions may occur. Motta et al. [24] have examined the IoT-related literature to find twenty-nine definitions of the concept. Connectivity, a component of QoE, is among the common concepts within the definitions Motta et al. distilled. From those twenty-nine definitions, they have identified seven key facets. These facets must be considered when engineering an IoT software system. They include:

- Connectivity includes the medium for things to connect to implement the IoT paradigm. Connectivity may be challenged by security concerns or the quality of service.
- Things include the number of heterogeneous tags, sensors, actuators, among other things. There exist challenges of maintaining the identities of these devices as well as managing their behavior.
- The behavior of IoT systems may include emergent behavior, which is the nonobvious side effects resulting from the composition of individual parts into a system. The main cause of emergent behavior is due to the complexity of systems and the human interaction within them [25].
- The smartness of the things within the IoT system relates to how devices are managed, orchestrated, and their allowance and use of autonomous behavior.
- Problem Domain may refer to the industry or specific problem that the IoT software system is built to alleviate.
- Interactivity is not limited to the interaction between things and humans, but also the interaction amongst things within the IoT system. This implies the importance of interoperability.
- The environment is the context in which an IoT system operates and can also be specific to the problem domain or implementation.

## Methodology

### Data acquisition and preprocessing

**Fig. 1:**
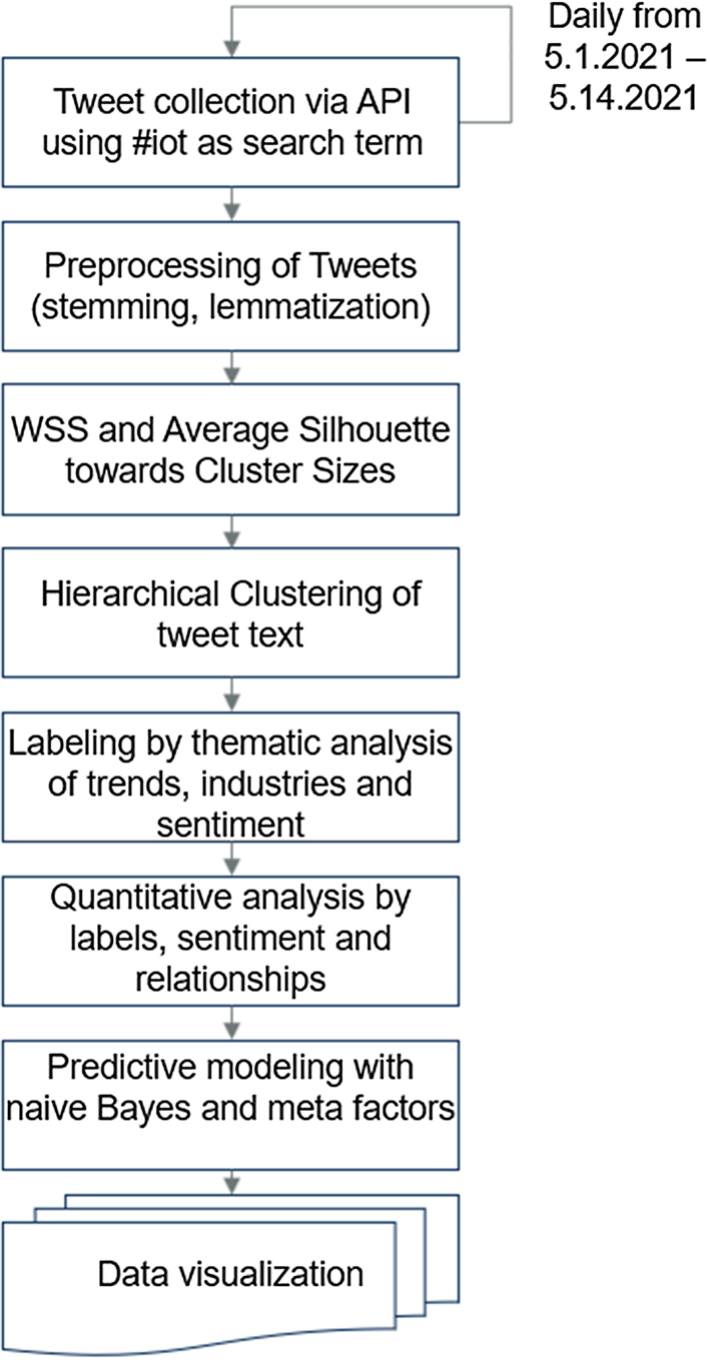
Seven steps make up the methodology starting with an iterative collection of tweets through labeling and analysis until the visualization of the data

We wrote and utilized an R program to manage the downloading of tweets from Twitter’s application programming interface (API). Another R program was created to label the tweets and to perform the content-based analysis. The analysis begins with preprocessing the tweets including the removal of stop words and usage of word stemming and lemmatization. The analysis includes an identification of trends within IoT discussions. The tweets are labeled for the factors of popularity (tweets that were liked or retweeted), industry mention, commercial vendor technology mention, and trend identification. There is an evaluation of sentiment within the labeled tweets. We also analyze the relationships between the factors of industry and trending terms. A naïve Bayes model is created to determine whether our labeled factors can predict the content or popularity of the tweets. Using the factors of favorite, industry type, retweet, and IoT vendor name, we could predict the trend a tweet was referencing with an accuracy of 63.9%. Figure 1 presents our methodology in seven steps. The seven steps are carried out in two R programs. The R programs and a compressed CSV file of the 684,503 tweets are available for use and evaluation on a publicly available Gitlab site [26] (Fig. 1).

To perform the collection of tweets, we first created a programming account on the Twitter platform. This account creation offered the authentication and authorization needed to access the Twitter platform via API. For the first fourteen days of May 2021, we searched for tweets containing #*iot* and stored up to 50,000 per day. The search limit was required as our AWS EC2 server instance is limited to four cores and 32 GB of memory. The impact of the limited server resources will be described later in this section. By the last day of tweet collection, we had successfully captured 684,503 tweets containing #*iot*.

### Number of cluster determination

After data collection, we created a document-term object matrix. The individual words from each tweet were then cast into the matrix and their frequency of appearance recorded. To determine an ideal number of clusters, we utilized within-cluster sum of squares (WSS) and the average silhouette methods. However, due to the size of the term matrix as input into these methods and the restrictions of our compute environment, only samples of the entire tweet corpus were used to generate the term matrix.

**Fig. 2:**
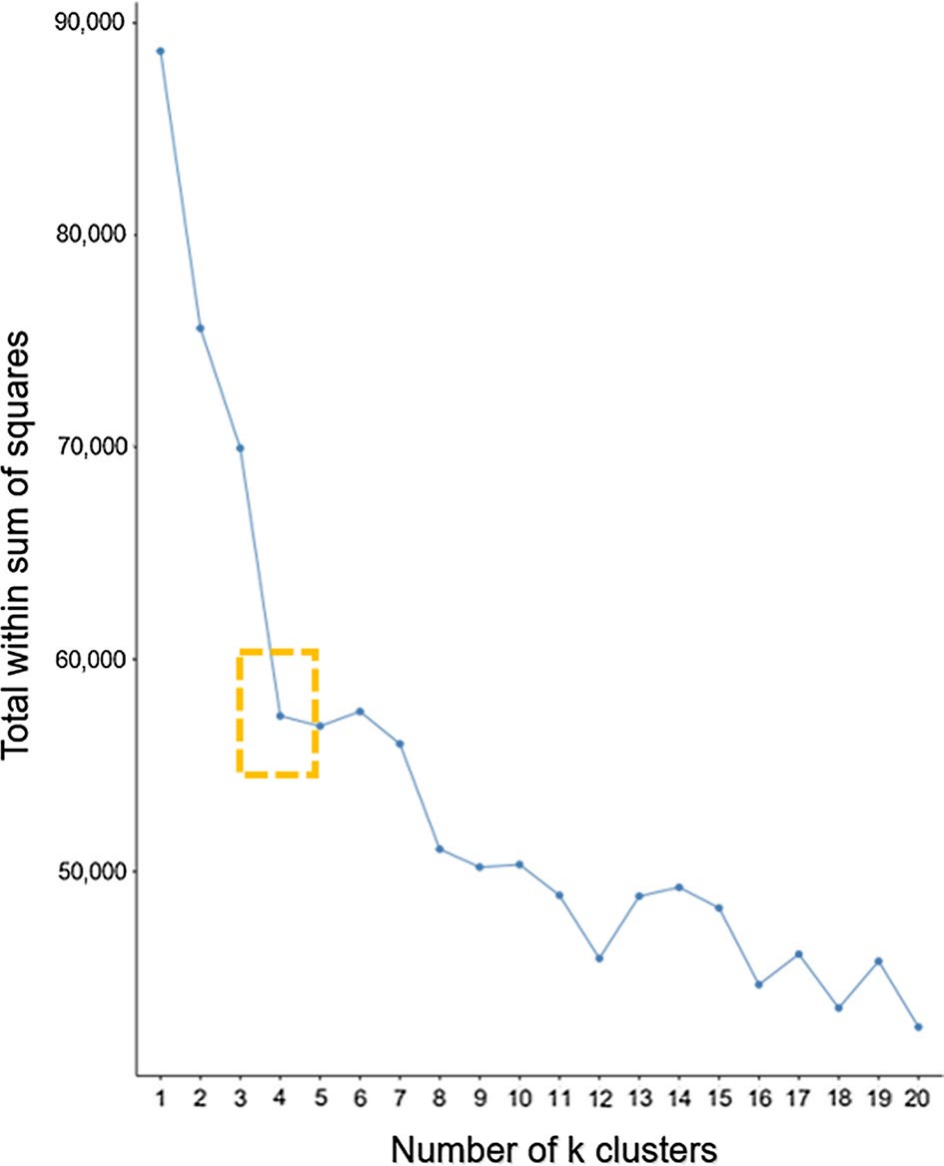
Output of the within-cluster sum of squares method to determine the proper number of clusters to be generated. The method indicated that four clusters were appropriate for the IoT tweets dataset

The WSS method will iterate through many generations of k-means clusters. During each iteration through k number of clusters, the squared distance between a cluster’s observations (within cluster) and the clusters’ centroid are summed and plotted for the given number of clusters. This is done for all clusters and compared for Euclidean distance over the iterations. The ideal number of clusters is frequently determined visually, known as the “elbow method” and identified when the WSS is decreasing and the next increment in cluster generation does not offer much benefit. This is often visually detected by looking for the “elbow” or the “knee” in the line chart where the WSS has dropped and then flattens. Figure 2 identifies the knee at four clusters for our dataset of #iot tweets collected over 2 weeks.

The silhouette method is like the WSS method in that it also generates many iterations of clusters and evaluates them for a proper *k* size. The average silhouette evaluation is performed by comparing the silhouette width of each cluster within an iteration to cluster widths of succeeding iterations having incrementing numbers of clusters. Overall, when many clusters are found within a small dimension, the width of the clusters (silhouettes) are smaller than if one cluster was occupying the same space. Thus, when having many small clusters in a dimension that could be optimized by having fewer clusters, the average silhouette method will indicate a small average cluster width and an improper number of *k* clusters.

**Fig. 3:**
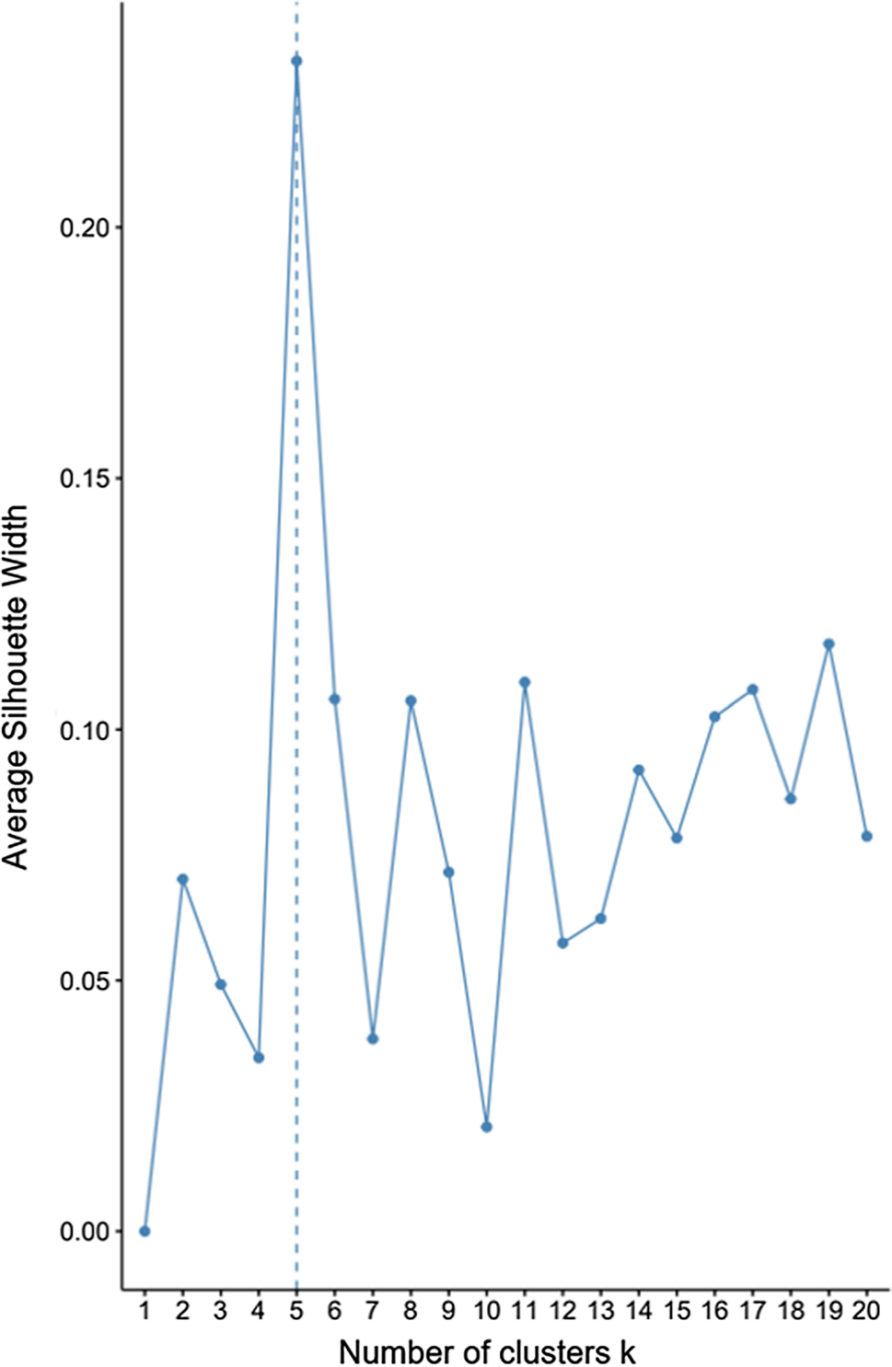
Output of the silhouette method to determine the proper number of clusters to be generated. The method indicated five clusters as appropriate for the IoT tweets dataset

Additionally, if clusters are generated as tightly grouped neighbors, then one observation in one cluster will be very close in distance to an observation in a neighboring cluster. The closeness of observations belonging to different clusters can indicate that the model suffers too many clusters. A quality number of clusters to generate would be the number of clusters that optimizes the largest average silhouette width. Ribeiro et al. [27] utilized maximum silhouette scores in their graph-clustering algorithm to identify groups of terms and their semantics. Their method, and the inclusion of silhouette scoring, outperformed previous methods. In our research, the silhouette method suggested the proper number of clusters for our dataset of IoT tweets to be five (as shown in Fig. 3), whereas the WSS method suggested the proper number of clusters to be four. To ease the execution of algorithms, we utilized R packages *factoextra* and *NbClust*.

**Fig. 4:**
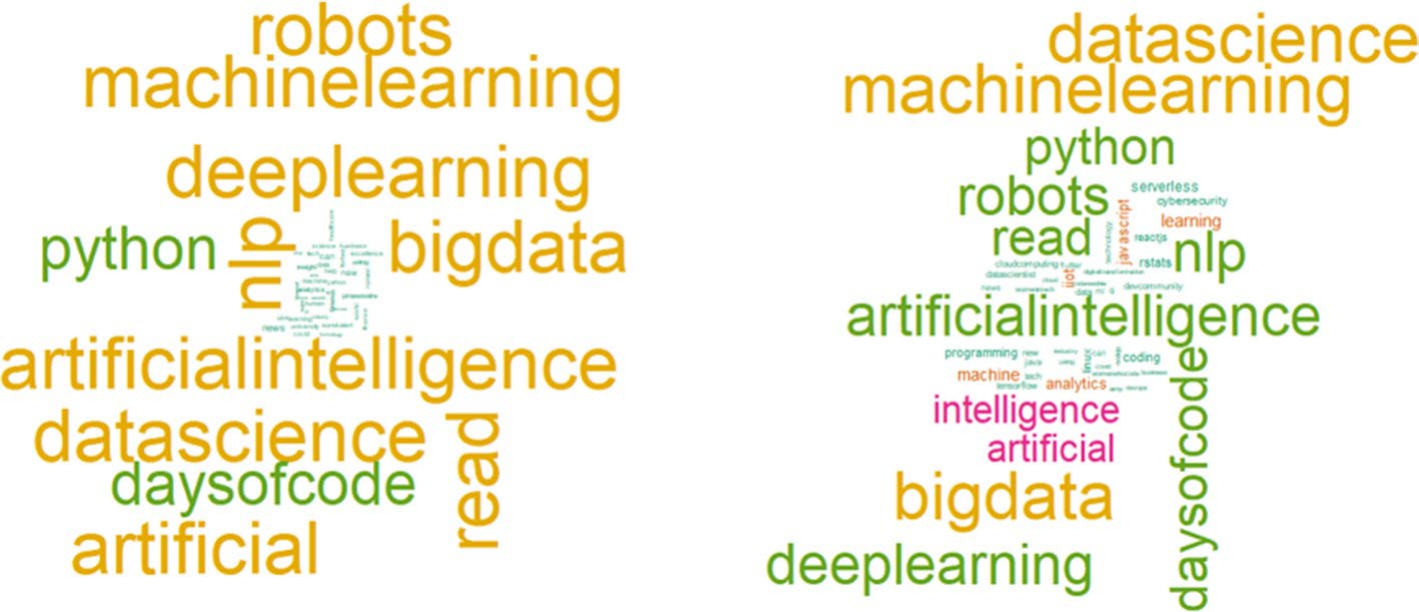
The leading trends do not include cybersecurity terms nor greatly shift whether four or five clusters of our IoT tweets were generated as indicated by word clouds of the largest clusters

Unsupervised hierarchical clustering was performed for both four and five cluster outcomes. An agglomerative method was used. With agglomerative clustering, each observation initializes as its cluster and through iterations is joined with nodes being the shortest distance away [28]. The difference of trend identification between the different cluster generations, whether four or five clusters, was not found to be interesting. This is further illustrated within Fig. 4 where the largest clusters of tweets were cast into word clouds. It is seen that the leading terms are still quite similar despite the differing number of clusters generated. What was most concerning, whether four or five clusters were generated, was the lack of any cybersecurity topic as a trending top ten topic. Only 12% of the 684,503 tweets contained any term related to vulnerabilities, hacking, malware, and other cybersecurity-related terms.

The tweets were labeled for having inclusion to industry, trend, and commercial vendor technologies. To determine industry names and search terms, we utilized a list by the International Labor Organization [29]. The tweets were also evaluated for their sentiment by utilizing the NRC lexicon [30]. Our analysis will be further discussed in the following section.

## Findings and discussion

### Unsupervised hierarchical clustering and top trends

Because the WSS and average silhouette methods identified the proper number of clusters for our dataset as four and five respectively, we generated clusters of tweets for both findings. However, the leading trends identified did not vary between four and five clusters as illustrated in the word clouds below. Word clouds are basic and intuitive tools that allow us to evaluate text results for insight [31].

**Fig. 5:**
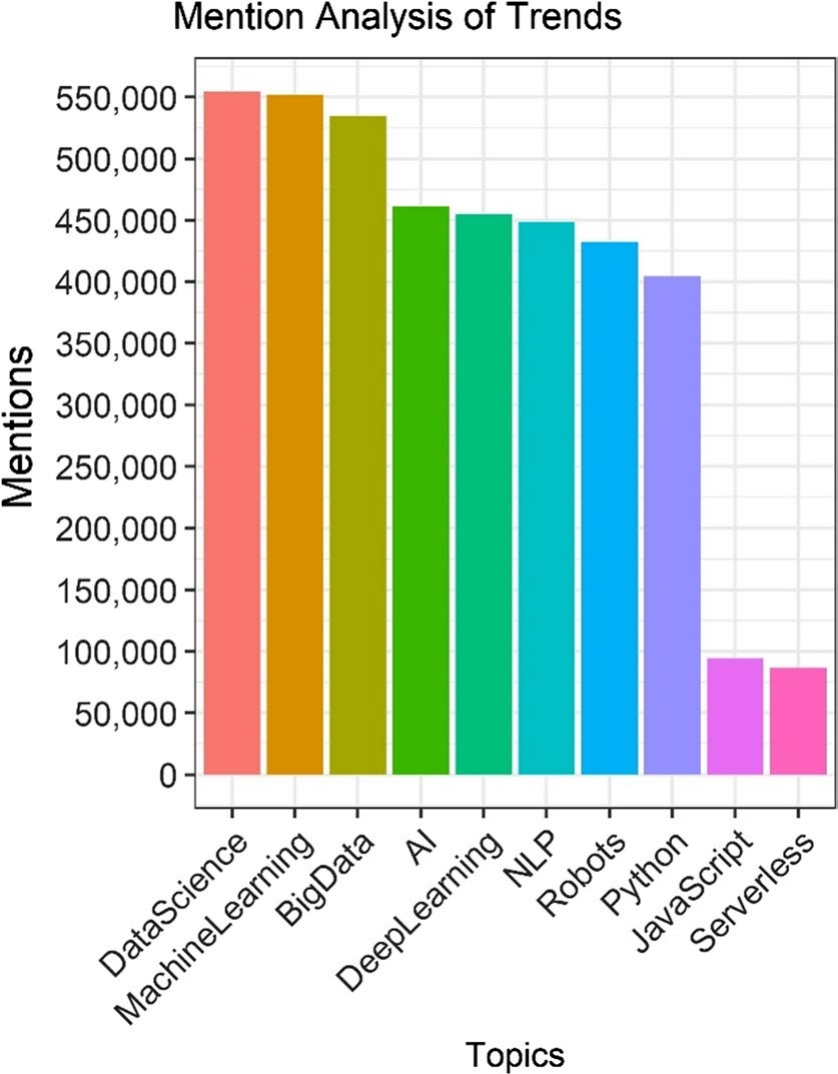
Term frequency is highest for data science, followed closely by machine learning

The word cloud on the left is the largest cluster when only four clusters were generated. The word cloud on the right is the largest cluster when five were generated. We performed a similar trend analysis throughout the cluster creation and the leading identified trends did not alter. Regardless of the number of clusters created, the top mentioned term continued to be “data science”. It was closely followed by “machine learning”, and subsequent frequent terms began dropping off in mention at a greater pace than compared to the first and second most mentioned terms. The mention analysis of trending topics is illustrated in Fig. 5.

### A small number of cybersecurity mentions within the IoT tweets

**Fig. 6:**
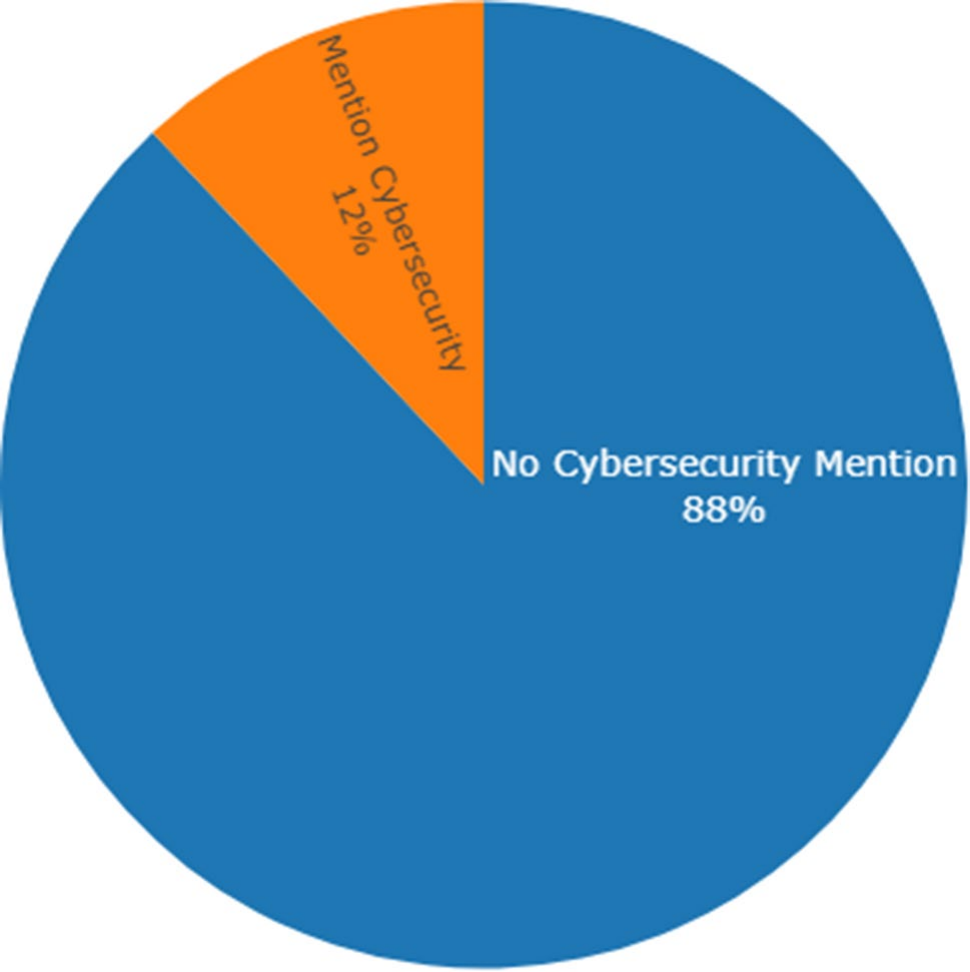
Only 12% of the total collection of IoT tweets had mention of common cybersecurity terms

Among the trend analysis, in general, what was most concerning was the lack of cybersecurity topics in the list of top mentioned terms. As illustrated in the following pie chart, only 12% of the 684,503 tweets had any mention of the following stemmed cybersecurity-related terms: cyber, secure, hack, vulnerability, risk, exploit, breach, malware, virus, ransomware, spyware, worm, trojan, encrypt or phishing (Fig. 6).

When tweets did mention cybersecurity terms, the topics of the three most retweeted conversations included an industry roundtable discussion [32], a reference to an opinion article about the risk of AI on military technology [33], and a reference to an article on the risk of AI on national security [34]. Among the most retweeted tweets discussing cybersecurity, the top three are each a technology being touted to secure IoT implementations.

### An IoT cybersecurity communications scorecard

The absence of frequent cybersecurity discussion within the collection of IoT tweets motivated examining which industries are communicating about risks the most. To compare the cybersecurity posture of industries based upon the public discussion found within our collection of tweets, we propose a new IoT Cybersecurity Communication Scorecard. The Balanced Scorecard was introduced by Kaplan and Norton in 1992 and links an organization’s broad performance measurements in four key areas [35]:

- customer perspective
- internal perspective
- innovation and learning perspective
- financial perspective

The purpose in a balanced scorecard is to align the organization to the strategy in areas such as human capital, information, and the organizational areas of culture, leadership, and teamwork [36]. A good cybersecurity scorecard helps improve the information and communication regarding cybersecurity [37]. Organizations have cybersecurity goals to be within compliance, protect their business, and to maintain their employees’ and customers’ trust. Cybersecurity is not just about technology and systems, but also the people and processes that rely on and are a part of the systems [38]. Our scorecard allows organizations to compare their communication of cybersecurity knowledge, awareness, and training to a benchmark of public discussion within their industry.

Our IoT Cybersecurity Communication Scorecard assesses posture by comparing the z-scores of density mention and sentiment scores to the relative averages of all collected tweets. Mention density is the percentage of all IoT tweets that mention cybersecurity topics. The mention density and sentiment are each normalized by mean and standard deviations into Z-scores. The z-scores reflect an industry’s position in terms of their cybersecurity mention density and the average sentiment of all tweets that reference their industry. The z-scores are found by first determining the average percentage of cybersecurity conversations among all tweets and the average sentiment of all tweets. The standard deviations are also recorded. The z-scores identify the positive and negative distance to the population’s mean. The posture score is a combined index of the two z-scores. We gave equal weight in the overall posture score calculation. If an organization placed significant importance on either the volume or the sentiment of the messages, they could apply custom weights.

Organizations should utilize the scorecard as a benchmark to compare their cybersecurity communication volume and sentiment to their industry’s scores. For an organization to utilize this scorecard as a benchmark, they must determine their mention density by dividing the number of corporate cybersecurity communications by the total number of corporate communications and compare to their industry within Table 1. A similar comparison can be done to understand the positivity and sentiment of their corporate cybersecurity communications.

**Table 1:** Industry cybersecurity scorecard by mention density and sentiment analysis

Industry	Mention density	Density Z-score	Sentiment score	Sentiment Z-score	Posture score
Mechanical	40.7%	2.187	0.041	− 0.033	2.154
Automotive	31.1%	1.384	0.024	− 0.194	1.19
Commerce	12.1%	− 0.198	0.119	0.699	0.501
Public	12.6%	− 0.157	0.077	0.31	0.153
Health	10.3%	− 0.353	0.093	0.461	0.108
Financial	5.4%	− 0.76	0.128	0.79	0.03
Media	2.3%	− 1.018	0.155	1.044	0.026
Transportation	7.6%	− 0.519	0.057	0.116	− 0.403
Agriculture	12.9%	− 0.13	− 0.038	− 0.783	− 0.913
Food	9.9%	− 0.38	− 0.21	− 2.412	− 2.792

The leading industry by posture score within this social media analysis was found to be mechanical. Tweets within the food industry scored the lowest posture. The food sector experiences pressures such as climate change, food price volatility and food security [29]. We must add cybersecurity risk to this list. Recently JBS USA Holdings, a food manufacturer which supplies the United States with roughly one-fifth of their meat supplies, experienced a public, expensive, and business impacting ransomware attack [39]. Due to the ransomware attack, JBS USA Holdings temporarily shut down operations in nine beef processing plants and eventually paid a ransom of $11 million [40]. Table 1 provides the density of cybersecurity messages and their sentiment by industry. The table is sorted by posture rank. The scorecard research is limited by only comparing the top ten industries by volume.

### Content-based analysis of industries within the IoT tweets

**Fig. 7:**
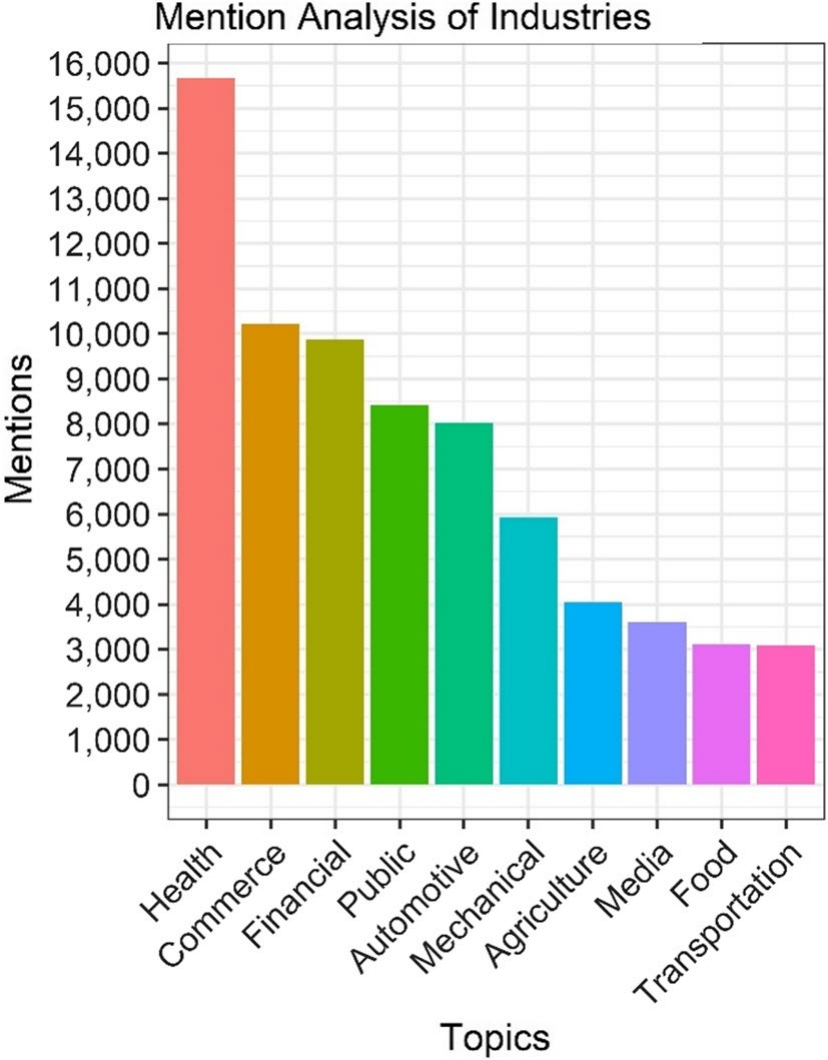
The top ten mentioned industry within the collection of IoT tweets was healthcare followed by commerce and then financial

What is further concerning by the dearth of cybersecurity-related discussions within the collection of IoT-related tweets is that the top mentioned industry was healthcare. Previous research identified healthcare as one of the lesser influential industries mentioned in research papers on IoT [41]. Our research and this paper are one effort in shifting that claim. The top ten mentioned industries are depicted in Fig. 7. It is not surprising to see healthcare leading the mentions as many countries are still experiencing the COVID-19 pandemic. While collecting these tweets based upon the inclusion of #iot, 4% of the tweets referenced COVID-19. Recent research has discussed the relationship between digital twins, IoT, and contact tracing technology [42], which could be utilized to help understand the behavior of a pandemic. After healthcare, the second most mentioned industry within the IoT tweets is commerce followed by financial.

### Network analysis and relationship identification

A network analysis was also performed on the relationships between trends and industries. Fundamental parameters of a network are its number of nodes, otherwise known as the network size, and the number of edges [43]. We are surrounded by naturally connected structures and networks [44]. Industries and technology trends are no different, as we confirm with this analysis regarding the health industry connections to all of the top identified IoT trends.

**Fig. 8:**
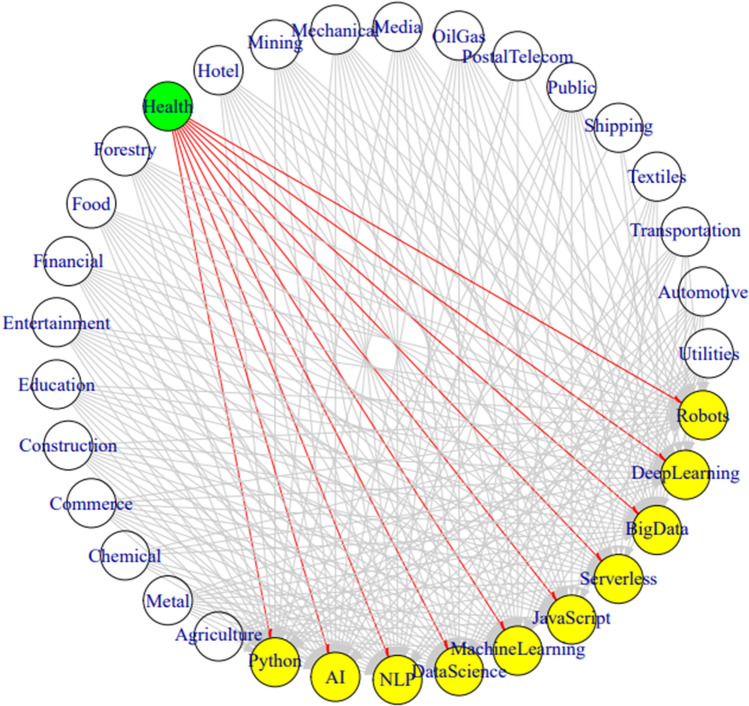
This network graph utilizes red arrows that depict relationships between tweets towards the healthcare industry, highlighted in green near the top of the image, and all of the trending terms which are lighted in yellow

To construct the network graph in Fig. 8, the tweets’ metadata labels were cast as nodes into two tables. The first table listed every industry and the trend terms (nodes) along with a unique identifier. The second table was a large list of the industry nodes, a corresponding trend node, and a weight column that indicated the frequency when a tweet was identified as matching both labels. Utilizing the network and igraph libraries in R, we plotted the node and edge relationships as the data visualization in Fig. 8. This figure is a network graph that has the most mentioned industry, healthcare, highlighted as a green network node. Then, red lines which indicate relationships, are drawn to each of the yellow trending terms given both labels co-exist in single tweet metadata that we created during our preprocessing. As the image indicates, all trend terms are found in the network of healthcare tweets. As Fig. 4 indicated, serverless was the least mentioned trending term, yet it too has an inner-tweet relationship to those tweets having reference to healthcare.

### Sentiment analysis of commercial technology providers within the IoT tweets

There are many technology providers which have solutions, offer services, or offer platforms to solve IoT opportunities. We performed a content-based analysis of technology vendors within the IoT space. To determine the list of IoT vendors to analyze, we utilized two 2020 research reports by Gartner [45, 46]. We utilized the sentimentr library to determine the sentiment scores of industry technology providers.

We plotted the technology provider names into a chart having four sections. The four sections of the chart have an x and y-axis, where the x-axis is the z-score of the tweet sentiments when the vendor is mentioned. The z-score is found by first determining the sentiment of all tweets that mention the commercial technologies, then calculating the average, and the standard deviation. Then, the z-score for a given technology vendor is calculated by dividing the commercial vendor’s mentioned tweet sentiment by the number of standard deviations away from the population’s average sentiment. The y-axis is measuring the number of times an IoT technology provider is mentioned in our corpus of tweets.

**Fig. 9:**
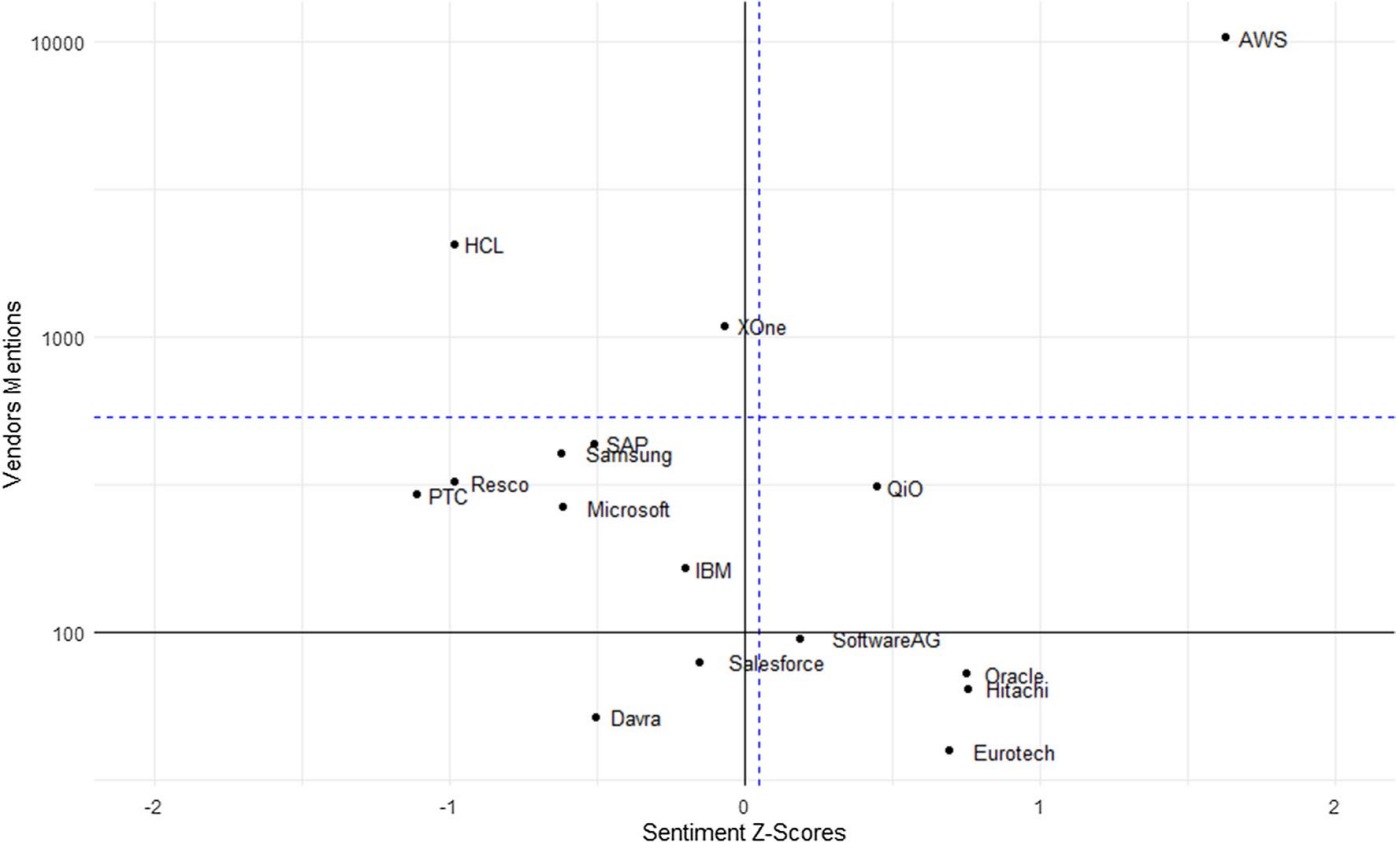
AWS has the most mentions and the highest sentiment among our corpus of IoT tweets while the technology company Davra would have a lesser number of mention and a sentiment less than average

In general, if a vendor is placed on the upper right area of the chart, that implies that they are widely mentioned and the sentiment of the tweets that they are mentioned within is above average sentiment. If a vendor is found on the bottom left side of the chart, they would be both lower in popularity and lower in sentiment positivity within this collection of tweets. Any vendors having less than ten mentions within the tweets were removed from the plot. The dashed blue lines represent the average mentions and average sentiment scores. The average sentiment of all tweets mentioning these IoT solution vendors is slightly positive. Use caution when reviewing the chart as the y-axis is intentionally logarithmic. The logarithmic axis allows the data to pull slightly apart, as though zooming in, for the vendors who have lesser mentions. The vendor placement can be viewed in Fig. 9.

Amazon’s AWS has the most mentions and the most positive sentiment among the vendors being mentioned within the IoT tweets. The AWS IoT Core can connect IoT devices to AWS cloud services and AWS offers an IoT SDK for development in languages such as Java, JavaScript, or Python. JavaScript was identified as one of the top ten trends in our analysis. AWS IoT Core product supports message brokering for these protocols [47]:

- Message Queuing and Telemetry Transport (MQTT)
- MQTT over Websockets Secure (WSS)
- Hypertext Transfer Protocol -Secure (HTTPS)
- Long Range Wide Area Network (LoRaWan)

Davra is within the bottom left area of the plot. They have fewer mentions in the analysis and the tweets that do mention them tend to have a lower sentiment than average across all of the analyzed technology vendors. Davra offers an IoT Platform that has features such as access control to both devices and services, service management features including edge, cloud, Kubernetes, or container deployments, as well as supporting many different IoT device protocols and data storage capabilities [48].

### Predictive modeling based upon our IoT tweet metadata factors

Naïve Bayes has been utilized to accurately forecast crime activities including arson, burglary, and theft [49]. Biology researchers have successfully applied naïve Bayes modeling to determine the presence of links in protein interaction networks, although anomaly detection was utilized to increase the accuracy [50]. In our research, we utilize naïve Bayes models to understand relationships between the IoT trends, the sentiment of the content, industries, and IoT technology providers.

Using a naïve Bayes model with a dependent factor of trend type and an independent variable of sentiment, we found that given a tweet is labeled as towards the trending topic data science, there is a 66.7% probability that the sentiment of the tweet is positive. Tweets that were labeled as towards the IoT trend of natural language processing (NLP) scored the second highest in positive sentiment probability at 57.1%. Table 2 notates the conditional probabilities as found by the model.

**Table 2:** Trending IoT tweet topics having the highest probability of positive sentiment are highlighted in this conditional probability table

Trends (below)	Anger	Anticipation	Disgust	Fear	Joy	Negative	Positive	Sadness	Surprise	Trust
AI	0.000	0.214	0.107	0.071	0.000	0.107	0.357	0.036	0.000	0.107
BigData	0.149	0.064	0.000	0.064	0.128	0.106	0.234	0.000	0.064	0.191
DataScience	0.000	0.222	0.000	0.000	0.000	0.000	0.667	0.000	0.111	0.000
DeepLearning	0.100	0.100	0.000	0.000	0.100	0.200	0.300	0.000	0.100	0.100
JavaScript	0.000	0.222	0.000	0.000	0.111	0.000	0.444	0.000	0.000	0.222
MachineLearning	0.045	0.136	0.000	0.091	0.000	0.136	0.455	0.000	0.000	0.136
NLP	0.000	0.143	0.143	0.000	0.000	0.143	0.571	0.000	0.000	0.000
Python	0.000	0.000	0.000	0.500	0.000	0.500	0.000	0.000	0.000	0.000

A second naïve Bayes model was created to help with understanding which factors influence the prediction of tweets being retweeted. The industry and trend factors had little impact on a tweet being retweeted. However, the sentiment did affect the probability of a tweet being retweeted. Given the tweets conveyed either fear or joy would improve the probability of retweet to 13.0% and 12.4% respectively. A third naïve Bayes model was used to predict which trending term an IoT tweet may be about. Using the factors of favorite, industry type, retweet, and IoT vendor name, we could predict the trend a tweet was referencing with an accuracy of 63.9%.

## Conclusion

There are new microblogs on the topic of the Internet of Things each day. From May 1st, 2021, to May 14th, 2021, we collected 684,503 tweets by searching Twitter’s API for #iot. While previous research has indicated that healthcare is not a top-three industry influence on the IoT [41], our research determined healthcare the most widely discussed industry in public IoT conversations on the Twitter platform. While the healthcare industry requires secured information systems, only 12% of the tweets within this IoT network analysis referenced cybersecurity concepts. Even less, only 10.3% of the healthcare related tweets referenced cybersecurity concepts.

From this collection of tweets, the most common trend term was data science. A network analysis graph depicted that every trending term was mentioned within healthcare-related tweets. Whereas for the tweets regarding the shipping industry, only the trends of AI, big data, and machine learning were related. IoT practitioners should utilize the network analysis to see how similar organizations are communicating and including technical concepts in their implementation.

No cybersecurity-related terms or concepts, such as encryption, ransomware, zero-trust, or vulnerabilities, were identified as trending terms. In general, there was an alarming dearth of conversations towards cybersecurity as only 12% of the IoT tweets contained any mention of cybersecurity related topics.

The trending terms having the highest probability of positive sentiment in a referencing tweet were data science followed by natural language processing. We could predict what trend a tweet was referencing with a 63.9% accuracy. To reach that level of accuracy in the model we utilized the factors of whether the tweet had been retweeted, marked as a favorite, and by knowing the industry and vendors being mentioned in the tweet’s text. IoT practitioners need to review our identified trends for how these technologies can benefit their implementations and end-users. Future research should include a comparison of the trends we have identified and how they may change over time.

A new IoT Cybersecurity Communication Scorecard was proposed. The posture was scored by the density of cybersecurity conversations and their sentiment. The top ten mentioned industries were ranked by their posture using our IoT cybersecurity communication scorecard. The mechanical industry had the highest rated posture. The scorecard is limited in that in only ranks based on communication regarding cybersecurity and future research is required to tie the posture score into the many additional areas of securing systems. IT security leaders should utilize this scorecard to benchmark their cybersecurity communication density and sentiment compared to the public discussions referring to their industry.

Amazon AWS that had the highest average sentiment among the vendors that were considered in this research. It was also Amazon AWS that was most frequently mentioned in this collection of tweets. Commercial firms can utilize our research and Fig. 9 to assess competing organizations and improve their social media presence and marketing messages.

A limitation of this research is that only one microblogging site, Twitter, was utilized for data collection. Another limitation was the available computing power of our systems. Our experience is that 32 GB of memory is not sufficient when analyzing 684,503 tweets and thus forces the use of samples within the collection. Specifically, we turned to use samples when carrying out the unsupervised hierarchical clustering and the naive Bayes models within our methodology. There is a need to study time dependent trends that will examine if the communication regarding cybersecurity is increasing towards acceptable values. Such research will require periodic data collection for a period spanning several months or a few years.

## Data Availability

The datasets analyzed during the study and the R code are available in the GitLab repository, https://gitlab.com/jimscheibmeir/socialmediaanalyticsofiot.

## Abbreviations

API: Application programming interface
CSV: Comma-separated values
DoS: Denial of service
IDT: Intrusion detection systems
IoT: Internet of Things
NRC: National Research Council Canada
NLP: Natural language processing
QoE: Quality of experience
WSS: Within-cluster sum of squares
